# Successful Treatment of a Three-Column Thoracic Extension Injury with Recumbency

**DOI:** 10.7759/cureus.614

**Published:** 2016-05-18

**Authors:** Winward Choy, Zachary A Smith, Stephanus V Viljoen, Timothy E Lindley, Nader S Dahdaleh

**Affiliations:** 1 Department of Neurosurgery, Feinberg School of Medicine Northwestern University; 2 Department of Neurosurgery, University of Iowa; 3 Department of Neurosurgery, Sanford Health

**Keywords:** thoracic fracture, thoracic extension injury, hyperextension injury, posterior ligamentous complex, ligament healing

## Abstract

We report a unique instance of a 66-year-old male patient with an unstable three-column thoracic extension injury at the level of T4/5 who was treated with recumbency and bracing without surgery. A posterior long segment fixation was attempted three times on two separate occasions over the course of a week with failure due to difficulty in ventilating the patient during prone positioning, cardiopulmonary arrest, and hemodynamic instability during prone positioning for surgery. The decision then was to treat this fracture with recumbency. He was fitted with a thoracolumbosacral orthosis (TLSO), and was kept on bed rest for eight weeks. The patient’s activity was advanced to head of bed for 45 degrees for four weeks and then to 90 degrees for four other weeks. At his 16th week visit, the patient was asymptomatic, and a computer tomography (CT) scan and magnetic resonance imaging (MRI) of the thoracic spine demonstrated evidence of osteophyte bridging and restoration of normal alignment. Three-column thoracic extension injuries can be successfully treated with recumbency in poor surgical candidates.

## Introduction

Extension injuries of the thoracic spine due to trauma are infrequent, [1] and typically result from hyperextension in combination with posteroanterior shearing force. Thoracic extension injuries can carry significant morbidity and often require surgical fixation for stabilization. We report a unique instance of a patient with a three-column thoracic extension injury treated with recumbency and bracing after multiple aborted attempts at surgical stabilization. An MRI at the 16-week follow-up revealed that the symptoms resolved with evidence of healing with osteophyte bridging at the level of injury. Informed consent was obtained from the patient for this study.

## Case presentation

**Figure 1:**
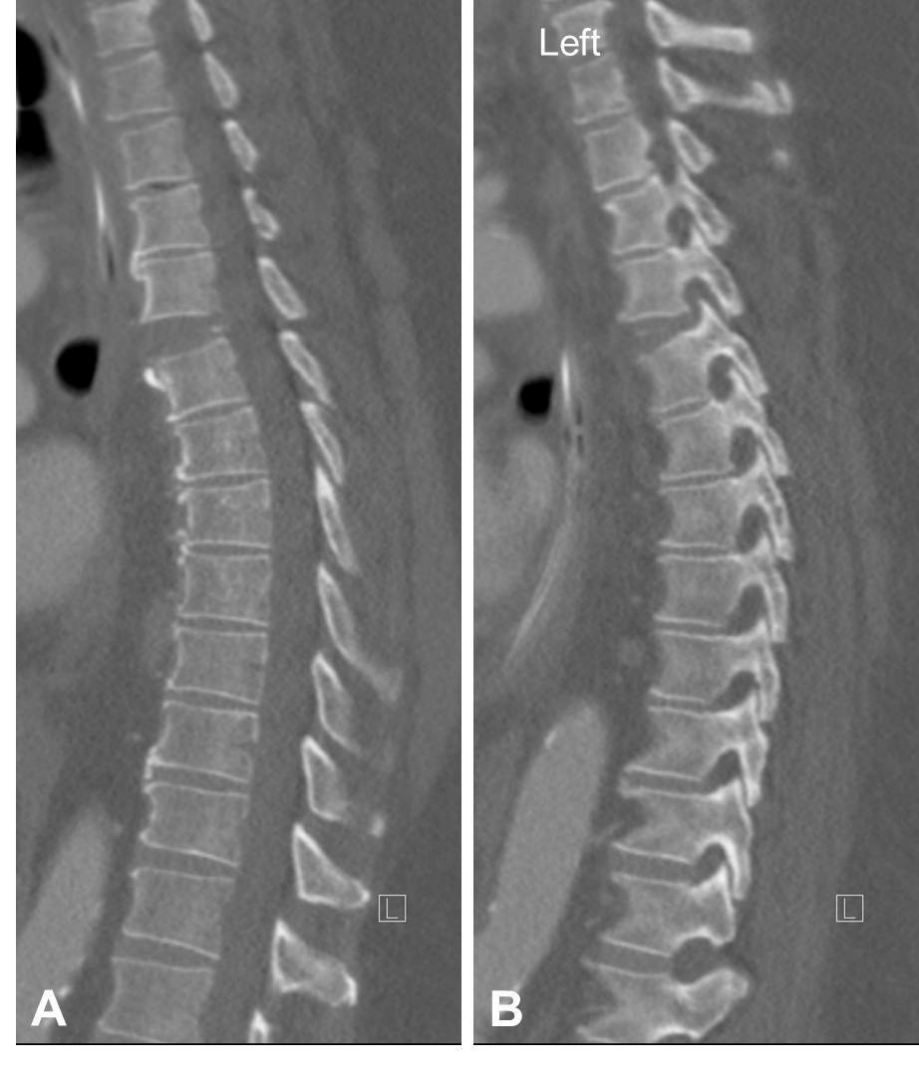
Sagittal Computerized Tomography (CT) of Injury A sagittal CT demonstrates extension type injury at T4/5 (A). A left parasagittal cut shows distraction of the facet joint at that level (B).

**Figure 2:**
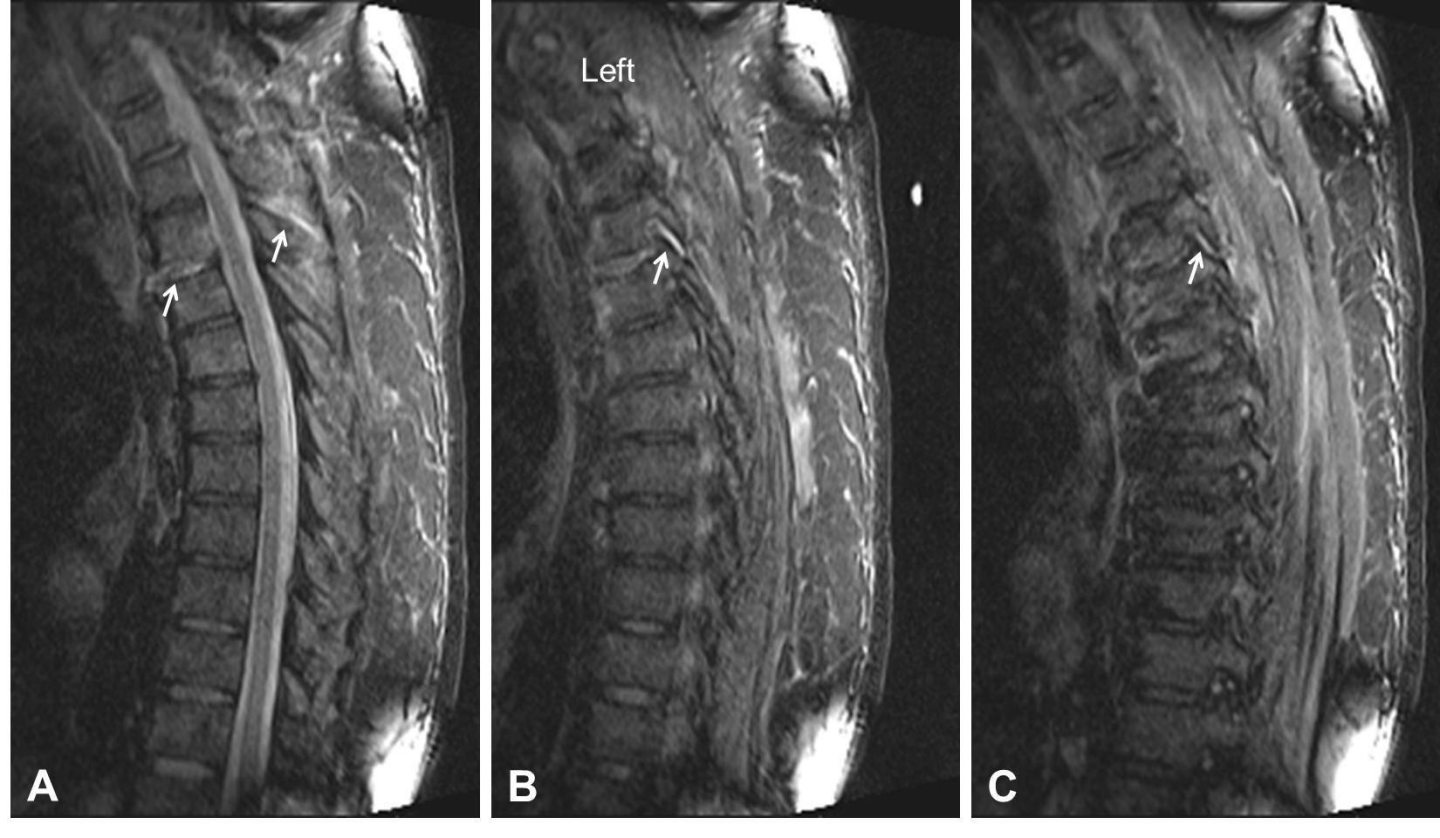
Magnetic Resonance Imaging (MRI) of Injury An MRI short tau inversion and recovery (STIR) sequence demonstrates high signal intensity at the level of the anterior longitudinal ligament, disk, and posterior ligamentous complex (interspinous and supraspinous ligaments) at the level of T4/5 indicating disruption (A, arrows). A left (B, arrow), and right (C, arrow) parasagittal image shows high signal intensity at the level of the facet joints indicating disruption.

A 66-year-old male suffered a tractor accident resulting in cardiac contusion, multiple rib fractures resulting in flail chest, and a T4/5 hyperextension injury in the setting of diffuse idiopathic skeletal hyperostosis (DISH) (Figure 1). His neurological exam was normal; however, MR imaging revealed an extension distraction injury with discoligamentous disruption at the T4/5 level (Figure 2). The patient was obese class III, with a BMI of 60. A posterior long segment fixation was attempted three times on two separate occasions over the course of a week, but both occasions failed. The initial attempt failed due to difficulty in ventilating the patient during prone positioning. The latter failed due to cardiopulmonary arrest and hemodynamic instability during prone positioning for surgery.

**Figure 3:**
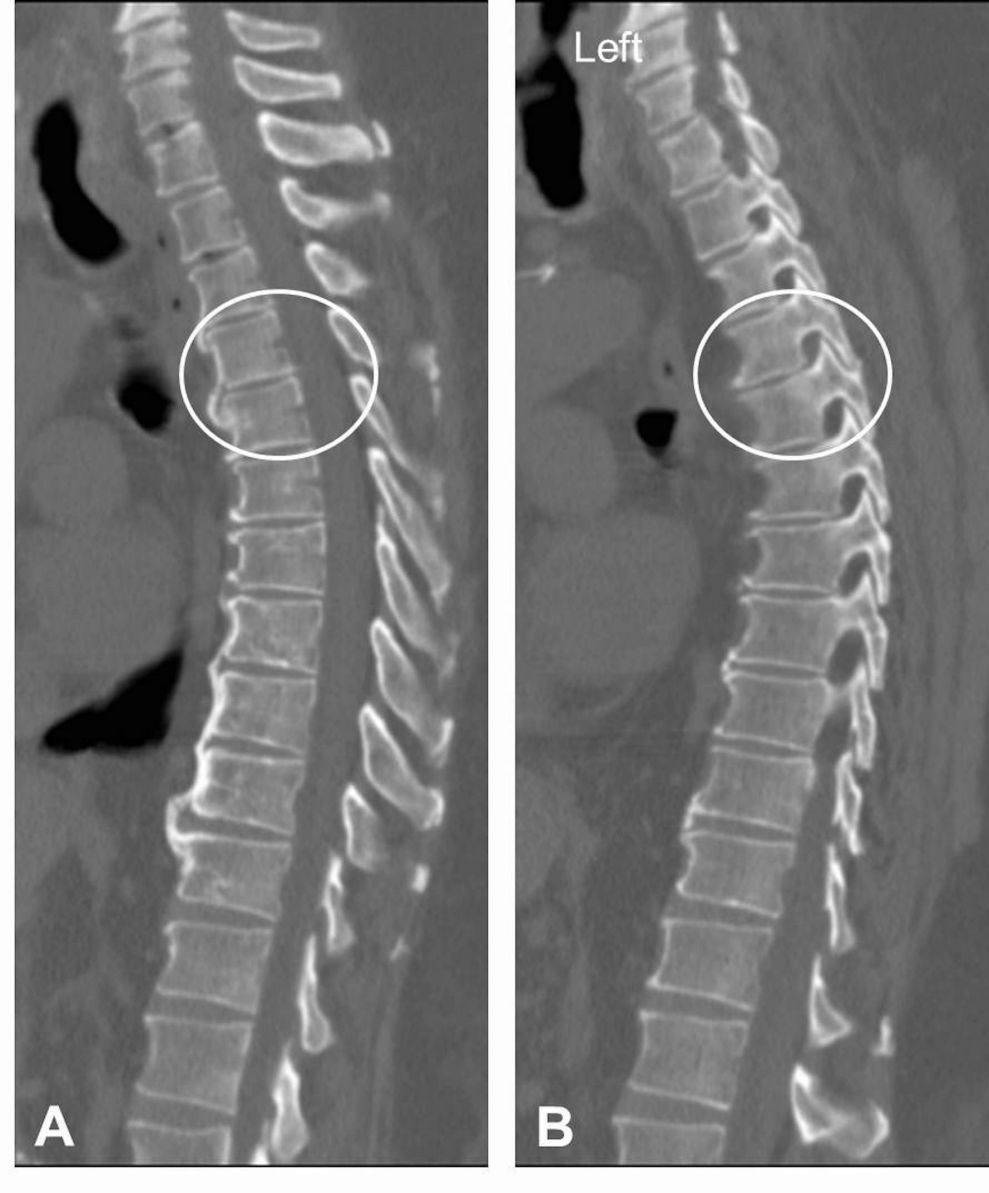
CT Following Recumbency Treatment A sagittal CT 16 weeks after recumbency treatment of the extension injury shows normal anatomic alignment of the thoracic spine as well as fracture healing with anterior osteophyte bridging between T4 and T5 (A, circle). A left parasagittal cut shows restoration of alignment and no distraction at the level of the facet joint (B, circle).

**Figure 4:**
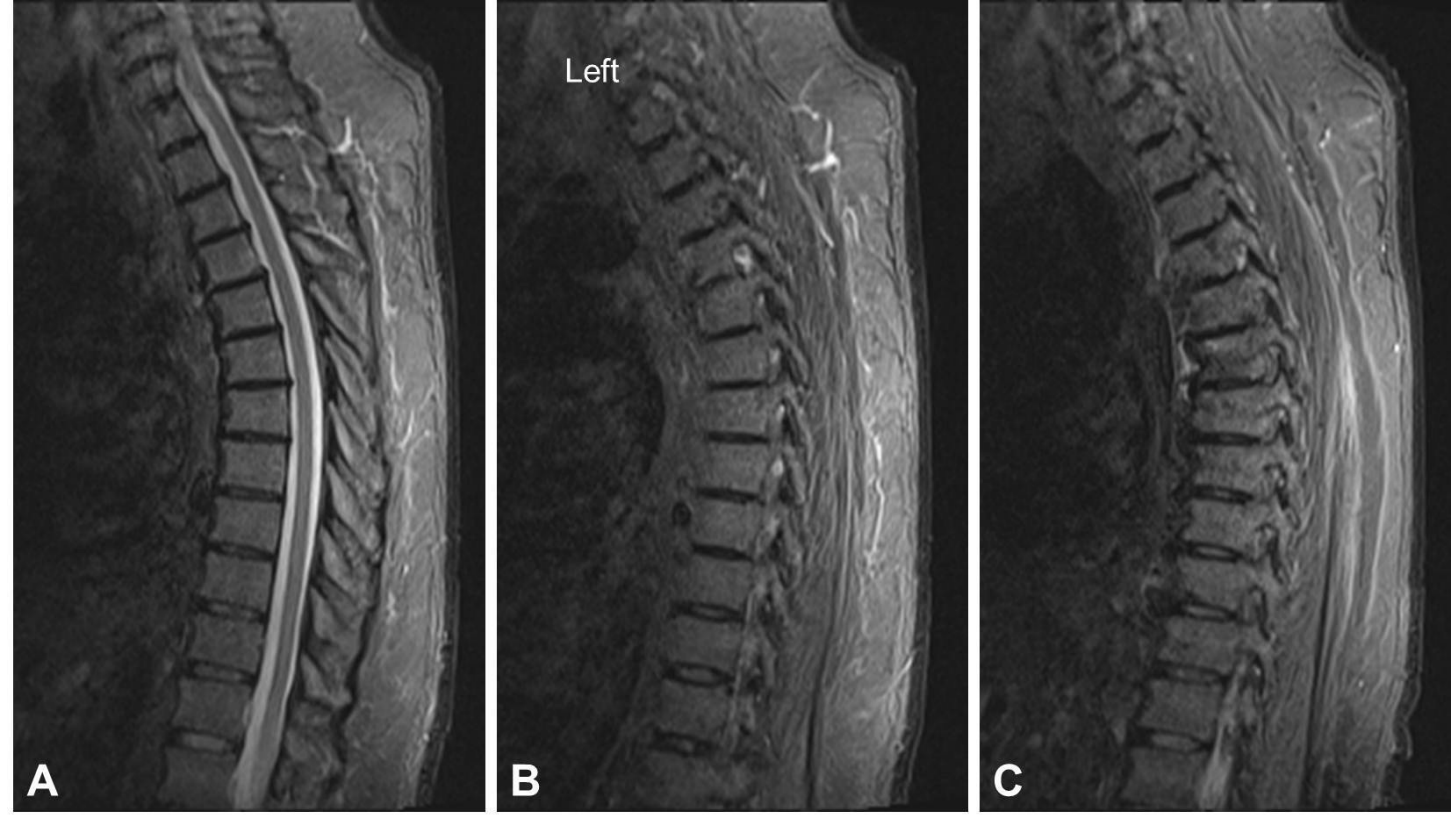
MRI Following Recumbency Treatment An MRI short tau inversion and recovery (STIR) sequence demonstrates normal signal intensity at the level of the anterior longitudinal ligament, disk, and posterior ligamentous complex (interspinous and supraspinous ligaments) at the level of T4/5 and bone osteophyte formation (A). A left (B), and right (C) parasagittal image shows normal signal intensity at the level of previously disrupted facet joints.

Due to high risk of surgical morbidity and inability to tolerate surgery, the decision was made to treat this fracture with recumbency. The patient received inferior vena cava filter placement and was started on prophylactic coumadin. He was fitted with a thoracolumbosacral orthosis (TLSO). He was kept on bed rest for eight weeks. The patient’s activity was advanced to head of bed of 45 degrees for four weeks and then to 90 degrees for four subsequent weeks with plane X-rays obtained at each visit confirming normal alignment. At his 16-week visit, a computer tomography (CT) scan of the thoracic spine was obtained demonstrating normal anatomic alignment of the thoracic spine as well as fracture healing with anterior osteophyte bridging between T4 and T5, and absence of distraction at the level of the facet joint (Figure 3). Moreover an MRI at 16 weeks demonstrated normal signal intensity at the level of previously disrupted facet joints (Figure 4). At that time the patient did not complain of any back pain and his neurological exam remained normal. Following that, the patient’s activity was advanced as tolerated and his brace was discontinued. On last follow-up visit he remained asymptomatic, and standing radiographs demonstrated normal alignment. 

## Discussion

Extension injuries often occur in the setting of ankylosing spondylitis and DISH [2-3]. These fractures involve all Denis three columns. In the setting of motor vehicle collisions (MVC), extension type injuries comprise only 6.8% of all injuries in which nearly all are located within the thoracic spine [1]. Concordant with the present case, extension injuries in the setting of MVC are associated with older age, higher BMI, and often present with concurrent chest injuries [1].

Similar reports by Elgafy et al. and Dr Oliveria et al. describe thoracic extension injuries that commonly result from a high velocity vehicular collision from behind [4-5]. The proposed mechanism of injury is a combination of hyperexention in the setting of a posteroanterior shearing force and disruption of the anterior longitudinal ligament. Although the reported degree of neurological damage was variable, these injuries are considered unstable and are often treated with long segment posterior pedicle screw fixation with the goal of bony union [4, 6].

According to the thoracolumbar injury classification scoring system (TLICS), if a posterolateral corner (PLC) injury is present, then three points are added to the fracture score signifying increased instability and requirement of internal fixation [7-8]. However, the patient in the present case was unable to tolerate multiple attempts at posterior long segment fixation due to poor ventilation, cardiopulmonary arrest, and hemodynamic instability during prone positioning. In this case report, we report the unique and successful treatment of an unstable extension injury with recumbency in a high risk surgical patient who could not tolerate three attempts of posterior fusion. At 16 weeks follow-up, MR and CT images demonstrated evidence of osteophyte bridging and restoration of normal alignment.

Indeed, changes in CT and MRI prior and after recumbency treatment might not reflect the extent of histological healing and the restoration of normal biomechanics across the fracture segment. Given that the patient had no back pain, demonstrated a normal neurological exam and normal spinal alignment, we could safely assume that he was at least clinically stable obviating further intervention.

## Conclusions

Thoracic extension injuries in MVC are uncommon, and commonly require surgical fixation for stabilization. However, select patients with significant comorbidities may be unable to tolerate surgery without risk of serious perioperative complications. This case uniquely illustrates that unstable three-column thoracic extension injuries can be successfully treated with recumbency, and may have a role in patients who are poor surgical candidates.
